# Analectic

**Published:** 1833

**Authors:** 


### ANALECTIC, ANALYTICAL, AND ORIGINAL.

### I. Analectic.

1. *On Nervous Ganglions, and on the Origin and Nature of the Intercostal Nerve.* According to Professor Scarpa, the ganglions consist entirely of divisions and subdivisions of the nervous filaments which enter into them; they are surrounded by a soft cellular tissue, and imbued with fluids, and these filaments re-unite again to pass out from the ganglions. The compound ganglions received filaments from different origins, the cords which pass out from them are necessarily formed of filaments coming from these same various origins.

The trunk of the intercostal nerve and its branches contain as many filaments as intercostal nerves which come from them, and also nerves of the fifth and sixth pairs; as to the sixth, it is not known whether it gives or receives. The intercostal nerve, to speak accurately, has no separate existence, but is formed by the junction of almost all the others. It is the same with the bracheal plexus; each branch which comes out, carries with it filaments of all those which enter it, (the inferior cervical, and first dorsal. The inferior branches of the intercostal are the most compound; thus in affections of the bladder, and of the uterus, they disturb the whole body. A question naturally arises, that since the intercostal receives filaments from all the spinal nerves, why is it not subject to the will? The difference observed in the firmness of its texture, compared with that of nerves of sense, is not a sufficient reason; and the effect which has been attributed to its ganglions, of intercepting the effects of the will, is neither proved nor probable; but the explanation of the fact is found in the confirmation which the opinion of Galen has lately received by the experience of M. Panizza, relative to the different faculties of the two roots of the spinal nerves. We now know, in fact, that the anterior roots give motion, and that the posterior, which have a ganglion, sensibility. It is the same with the cerebral nerves; and Mr. Charles Bell has shown that the section of the suborbital nerve, (branch of the fifth pair,) destroys the sensation of the lips, and of the nasal region, and that of the facial nerve, (coming from the seventh pair,) destroys the motion of the same parts.

The fifth pair is divided into two parts, the smaller, which is distributed to the muscles of mastication, the larger which is appropriated to the senses. The lingual branch of the latter is devoted to the function of taste; but it is the ninth pair which gives motion to the tongue, and to the os hyoides. M. Scarpa thinks, moreover, that the nerve of the eighth pair, upon which he has found immediately after it goes out from the cranium a ganglion, a character which he regards as appertaining exclusively to the nerves of sensation, could only be destined to give sensibility to those parts in which its filaments are distributed, whilst the accessory nerve could give motion to those which are of a muscular nature. Setting out from these facts, M. Scarpa has carefully examined from what roots the filaments, which go to the intercostals, arise; and he has discovered that these filaments invariably arise from the posterior. A little above the ganglion, these filaments, at first to the number of three or four, and which are afterwards united into one or two cords, pass above the anterior root, and sometimes envelope it, as in a net-work, or even traverse it. Attention and skill are necessary to distinguish these variations. Thus Schmidt is deceived in believing that the filaments of the intercostals come from the anterior root, they certainly arise solely from the posterior; for they originate before the re-union of the fasciculi arising from the two roots. It follows hence, that the fleshy fibres of the heart and of the stomach do not receive filaments from the nerves of motion, but from those of sensation. They owe their excitation to the blood and to the food, and not to the will.

In a second letter M. Scarpa returns to the character which he attributes to all the nerves of sensation, of having ganglions. The cerebral nerves have them, according to him, whenever they are sensitive. Thus the olfactory has its bulbous extremity. The large portion of the trigemini ganglionary at their origin, has also the ophthalmic, spheno-palatine, and maxillary ganglions. None of the filaments which pass off from it go to the muscles. The par vagum hardly passes from the cranium, when it swells out into a ganglion. "It might be objected," says M. Scarpa, "that the ophthalmic ganglion comes partly from the motor oculi; I deny this second origin; it comes from the nasal branch of the fifth pair; the little band which passes out from the branch of the motor oculi, destined for the obliquus minor, is a cellular, and not a nervous ligament. This nasal nerve also gives off ciliary branches below the ganglion, and the motor oculi does not give off any: thus the motion of the iris does not depend on the will."

One would be tempted to suppose, from what has been just said, that the muscles of the eye, by an exception of which they would be the sole example, receive only nerves of motion, and not of sensation; but M. Scarpa believes that they also receive the latter. He suspects that the abductor receives a twig of that which proceeds directly from the brain, and accompanies the nerve of the sixth pair. As to the others, they receive, according to this authority, their sensibility from the filaments, which, passing out from the superior cervical ganglion of the intercostal, turn themselves towards the eye following the course of the carotid artery, the ophthalmic artery and its divisions. But how is it, that nature, when the ciliary nerves are so near, should cause the former to originate from such a distance? It is a question which the author proposes without attempting to solve. The posterior roots of all the spinal nerves, after having passed beyond the ganglions of the sensitive roots, unite very intimately with these latter. The same union may there be seen on a small scale, as in the great plexuses. These are the sensitive filaments; which, disentangled from the end of the others, go to the skin to constitute the organ of touch. It is necessary then to abandon the idea, that the touch is exercised by the same nerves as motion.—(*Extract of two letters addressed by M. Scarpa to M. Weber.*)

2. *Case of an Eruptive Disease arising from the use of Cubebs.*—John North, Esq. relates in the *London Medical and Physical Journal*, for March last, the two following cases, in which an eruptive disease was produced by the use of cubebs.

"Case I. Capt. P. after trying various remedies for the cure of an obstinate yet trifling gleet, took, of his own accord, large doses of cubebs three times a day. On the fourth day from the commencement of the remedy, he complained of great restlessness, flushing of the face, sickness, and head-ache. On the fifth, he was covered with an eruption from head to foot, which was accompanied by a high degree of sympathetic fever. The peculiar appearances of the eruption I will attempt to describe in the next case, which was more severe, and attended by more strongly-marked characters. Captain P. recovered in a few days, by the assistance of mild aperients, salines, and confinement to the house: he was, of course, desired to discontinue the cubebs. Upon a subsequent occasion, he again ventured to try the remedy, but he gave it up after taking one dose of it, in consequence of feeling the same general symptoms of restlessness, &c. which had preceded the former attack.

"Case II. Madame T. had long labored under severe leucorrhœa, and, from motives of delicacy, had neglected to consult any medical practitioner. She was recommended, by a friend, to try the effects of powder of cubebs, in doses of a small tea-spoonful twice a day. After the first two doses, she felt feverish, with a tingling and heat over the whole surface of the body; she had rather severe headache, and a very disagreeable sensation of fulness in the eyes; the hands and feet felt hot and numbed. On the third day, a 'flush of red' broke out over the whole of the body; and on the fourth, a decided eruption appeared The cubebs were now discontinued, six doses having been taken. On the fifth day, my attendance was required: her appearance was now peculiar, and rather formidable: her features were so completely changed, as to leave no trace of their natural expression; the face was excessively swollen; eyes turgid and watery; lips puffed, dry, and shining; coryza; hands much swollen, and so stiff that the fingers could not be bent; respiration hurried and difficult; pulse small, and varying from 120 to 130; skin burning hot; intense thirst; tongue very white; constant nausea, and occasional vomiting. She was covered with an eruption from head to foot, which, in some parts, could not have been distinguished from Urticaria febrilis, while in others it had more the appearance of lichen in its papular stage, but with much more intense inflammation around the base of the papulæ than is commonly seen in that disease. A fortnight elapsed before this lady completely recovered. The remedies employed, consisted of purgatives, salines, and mild anodynes at bed-time."

Mr. North says, that several other cases of the same kind, but milder in degree, have fallen under his care; and a very striking case is recorded in the *Archives Generales*, for Novomber, 1831. A young man was admitted into La Pitie under M. Velpeau, with gonorrhœa, which had existed for two months. After a few days he was ordered a mixture containing cubebs, copavia, and magnesia, in the proportions of two drachms of copavia to four of cubebs. He took this mixture daily, and on the sixth day he was attacked with violent itching and burning over the head and neck. In the morning, his face was covered with dark red patches, and in the course of a few hours, this appearance of the skin, as well as the itching and sensation of burning, had extended over the chest and arms. The following day the abdomen was attacked in the same manner. On the third day, the efflorescence that had at first appeared, became pale, but the legs and feet now presented a similar appearance. The case might easily have been taken for measles, if the cutaneous symptoms only had been regarded, but there was none of the characteristic constitutional disturbance, that attends that disease: the general health was, in fact, undisturbed. In the course of a few days, the eruption entirely disappeared; and if any doubt had existed as to its cause, it was completely removed by the following circumstance: about three weeks after his recovery, the patient repeated a dose of the medicine, which he had accidentally kept, the eruption appeared on the following morning, and remained for two or three days.

A similar eruption is sometimes occasioned by the use of balsam copaiva; and two or three cases of the kind are related by Dr. Hewson, in the 5th vol. of the North American Medical and Surgical Journal.—*Am. Med. and Surg. Jour.*

3. *Treatment of Gastrodynia of* Dr Graves.—Sept. 1828. Rev. I. D——y, æt 36, rather corpulent, of temperate habits, has suffered for three years from violent attacks of gastrodynia. They last sometimes for forty-eight hours, and during their continuance, the agony is described to be so great as to make even death a desirable boon. The symptoms during an attack are—sensation of straitness in the stomach, with exquisite unintermitting pain, the pain shooting into both hypochondria; dry retching, and rejection of every kind of food and medicines; the countenance, during the attack, pale and expressive of distress. He is not able, (although his sufferings make him very attentive to this point,) to trace any connexion between his complaint and different kinds of food. The attacks usually seize him in bed, and sometimes set in at once with great intensity, at other times commence with comparative mildness, progressively increase in intensity, and then disappear gradually in the same manner as they came on. He is rendered somewhat more liable to an attack by confinement of the bowels, and feels relieved after full evacuations; the agony suffered until these are procured is extreme, and even then the relief comes but slowly, and always leaves an internal soreness about the epigastrium, which continues with very little diminution for weeks, and is always proportionate to the previous intensity of the pain. The average frequency of the attack is about once in every two or three months. He resides in the country, but during his present visit to town, he has had one of his attacks, which gave me an opportunity of seeing him in it. It came on late in the evening, without any apparently assignable cause, the pain, as he had in his previous account of some of the fits described it, being at first slight, but steadily increasing in intensity, accompanied with great anguish, rejection of every thing from the stomach, and frequent retching; the eyes were watering, countenance pale, and expressive of great suffering. The previous history leading me to view the disease as arising from morbid sensibility of the nerves of the stomach, I had determined, should an opportunity offer while the patient was under my immediate observation, to exhibit opium. I should perhaps have observed, that all the preceding attacks had been treated in the country by bleeding or leeching, purgative medicines and enemata. I gave him fifteen drops of *tinct. opii.* in half a glass of tepid water, without an admixture of any of the stimulants or aromatics, as ether, fœtid tincture, &c., which are frequently, but, I believe, injudiciously, in many cases added to opium, under the supposition of increasing its virtues. In a few minutes after taking the draught, the stomach became settled, the pain was checked in its progressive intensity, and soon after began to die away, leaving behind it a very trifling soreness, which had nearly quite disappeared on the following morning. This was the first occasion on which he had passed through an attack in this manner.

From the date of the above observations, Sept. 1828 to July, 1831, I had frequent opportunities of seeing this gentleman, and he never, during that space of time, experienced any symptom of relapse. Since July, 1831, I have not seen him, but I may be almost certain, from his silence, that he has continued to enjoy the same immunity up to the present May, 1832.

Concerning alkalies I may remark, that the liquor potassæ causticus, although not so well suited to be used as a domestic medicine, is much preferable to magnesia in removing acidity and heart-burn.—*Lond. Med. and Surg. Jour. July*, 1832.

4. *On the Treatment of Habitual Constipation.* By Robert J. Graves, M. D., &c.—In many chronic diseases, and in habitual constipation, it is of the greatest consequence to procure daily and regular discharges from the bowels. *Lavements* effect this purpose most conveniently, and possess the advantage of not interfering with or weakening the digestive functions of the stomach and upper portion of the alimentary canal. Many persons, however, particularly females, have an insuperable objection to this method of obtaining relief, and acquire the habit of taking aperient medicines whenever their bowels are confined

Various causes have combined to render blue pill and calomel almost popular remedies, to which many have recourse when their bowels are irregular, or the stomach out of order. Indeed, it is quite incredible what a number of persons are in the habit of taking these preparations, either by themselves, or combined with other purgatives, whenever, to use the common expression, they feel themselves bilious. This habit sooner or later induces a state of extreme nervous irritability, and the invalid finally becomes a confirmed and unhappy hypochondriac; he is, in fact, slowly poisoned, without the more obvious symptoms of mercurialization being at any time produced.

It is almost unnecessary to observe, that although saline aperients give temporary relief, they afterwards increase the tendency to constipation, and weaken the stomach. The class of purgatives least liable to objection consists of magnesia, aloes, rhubarb, colocynth, &c. for exhibiting which, many well known and excellent formulæ are used. But even these substances, whose debilitating effects on the stomach are not near so great as that of mercurials and salts are attended with the disadvantage of being required in larger doses in proportion as the bowels become accustomed to their action. To remedy this evil, Dr. Elliotson has suggested a valuable combination, consisting of compound extract of colocynth with minute doses of croton oil. This I have frequently given with the best effects: but it is liable to a serious objection, for unless the croton oil be perfectly mixed with the mass, some of the pills may be too powerful, while the others are comparatively inert, and consequently the patient is exposed to the danger of hypercatharsis, as I have twice witnessed, although in both cases the medicines had been prepared in the shop of a respectable apothecary. The following combination will in general serve to obviate costiveness, without diminishing the appetite, or being attended with the necessity of the dose being increased as the patient becomes accustomed to its use:—R. Electuarii. sennæ, oz.ii.; Pulv. supertart. potassæ, oz.ss.; Carbonatis ferri, oz.ii.; Syrupi zingiberis, q. s.; Ft. electuarium. For the first few days I generally add about two drachms of sulphur to this electuary; but as soon as its operation has been established, the quantity of sulphur may be diminished one-half, and at the end of a week it may be omitted altogether. The dose must be regulated by its effects, but in general a small tea-spoonful in the middle of the day and at bed-time will be sufficient.

The value of the carbonate of iron as a tonic aperient has not been duly appreciated; I have succeeded in curing with it alone, a practitioner of eminence in this city, who had been long subject to extreme constipation, and had been reduced to the necessity of taking an enormous dose of purgatives almost every week.

When injections carefully administered with Read's syringe fail to remove obstinate constipation, which they will sometimes, though rarely do, other means must be resorted to. Some practitioners are in the habit of giving one dose of active purgatives after another, adding to the strength of each dose in proportion to the obstinacy of the case. This is an imprudent and hazardous mode of proceeding. In such cases the stomach will generally be capable of retaining castor oil; and I prefer giving repeated doses of this medicine to any other when the bowels display such an unusual degree of obstinacy, inasmuch as it may be safely accumulated in the alimentary canal, and will in the end procure evacuations without any of the dangers which attend repeated doses of acrid or drastic substances. I generally commence with two ounces, to be repeated every second hour, until the desired effect is produced. I do not recollect who it was first made the important observation, that in obstinate constipation the first dose of castor oil must be large, but when this has acted on the bowels, the dose may be gradually diminished, provided that the medicine is continued every day for some time. I have verified this in private practice, and lately had a patient in the Meath hospital, whose bowels had resisted injections and the strongest cathartics. Three ounces of castor oil continued for two days in succession, two ounces on the next day, and one ounce on the fourth, were found quite effectual. In some, the daily dose may be thus gradually diminished to a tea-spoonful at bed-time.

When the tendency to constipation is habitual, and the patient is not effectually relieved by the daily use of injections, and when the peculiar circumstances of the complaint render the administration of aperient medicines by the mouth inadmissible, great advantage may be derived from the application of purgative liniments to the abdomen. The one I have found most useful consists of four parts of castor oil, and one part of tincture of jalap. This must be diligently rubbed into the region of the stomach every morning before the patient rises, and it must be done under the bed-clothes, lest the unpleasant odor should sicken the stomach. I am indebted to a medical friend for this suggestion, which I used with success in the case of a young gentleman, whose state had become almost hopeless.

In constipated habits, I have likewise occasionally derived very remarkable benefit from the use of nitric acid given in sufficient doses. It seems, like the carbonate of iron, to possess the advantage of combining tonic with aperient qualities.

In connexion with this subject, I may remark, that long-continued and repeated attacks of constipation, by enlarging the cæcum and colen, lay the foundation of other diseases. This happens most frequently in females, but is not uncommon among males. In such cases the enlargement of the guts may occasion either of two distinct forms of disease, both attributable to the retention and accumulation of hardened fæces. In one form the symptoms are calculated to mislead the medical attendant, by inducing him to believe that his patient is laboring under chronic hepatitis. Pain and tenderness, and in some, hardness, and even a degree of enlargement, are perceptible in the right hypochondrium, while the patient's aspect is bilious, and he not unfrequently complains of pains in the right shoulder. At times he is subject to violent fits of colic, or to what he compares to cramp in the stomach, particularly after the bowels have been confined, after eating vegetables calculated to degenerate flatulence, or after exposure to cold.

In the other form, the general health suffers less; the pain and other local symptoms referred to the right hypochondrium are not complained of, but the patient is occasionally subject, particularly on exposure to the action of the causes before enumerated, to violent attacks of vomiting and pain in the belly, which are accompanied by the characteristic symptoms of intestinal obstruction. The circumstance that the immediate attack was apparently induced by some palpable and known cause, such as an error in diet, or exposure to cold, may here deceive the practitioner, and cause him to overlook the fecal accumulation, without whose removal recovery cannot take place. I and two other practitioners were several times deceived in the case of a gentleman, of a robust constitution and great strength of body; and the true cause of the sudden and dangerous colics to which he was subject, was not discovered until he happened to mention, that when a young man, he seldom went to stool more than once a week. This led to the suspicion of an enlarged colon, and ever since the attacks have readily yielded to large injections administered by means of a Read's syringe, without which instrument he now never ventures to travel. The practical point that strictly claims our attention is, that the period of life at which the patient becomes subject to these attacks, is often long subsequent to the cessation or diminution of the habit of constipation, and consequently the physician will not perceive the true cause of the complaint unless he questions the patient very accurately.—*Dublin Journ. of Med. and Chem. Science.*

5. M. Dupuytren *on the Consolidation of Fractures of the Neck of the Femur within the Capsule.*—Sir Astley Cooper says, "that in all cases of transverse fracture of the neck of the femur within the capsule, which he has had occasion to examine, there never was seen an osseous callus." "The dissections which I have made," adds this illustrious surgeon, "have convinced me that the fragments of the fracture of the neck of the femur, when this happens within the capsular ligament, did not ever unite by an osseous callus; the re-union was made by a ligamentous substance only, as in fracture of the patella." Persuaded that the consolidation of the neck of the femur is impossible, Sir Astley has made experiments on living animals, which have confirmed him in his opinion. The English surgeons have equally adopted the opinion of their co-patriot. Baron Dupuytren, on the contrary, is of opinion that this consolidation may take place. A considerable quantity of anatomical pieces, he says, "representing intracapsular fractures of the femur, well consolidated, are to be found in the different museums. Those contained in the cabinet of the faculty of Paris, and of the amphitheatre of the Hotel-Dieu, really prove that this consolidation, with or without deformity, is real. Sir Astley Cooper has probably seen these fractures of the neck of the femur, which had not been cured, or which have been maltreated, or which have been partial. This is the only manner to explain the opinion of the English surgeon, which appears to us to be evidently erroneous. The examination of these pieces of pathological anatomy, is eminently proper to convince us of the reality of the consolidation of this intra-capsular fracture, does not appear, nevertheless, to have produced this effect on other English surgeons who have visited the museum of our faculty. Mr. Cross is said to have considered with care the pathological pieces preserved in the school of medicine of Paris, and not one among them did appear to him to be of a nature to prove that osseous re-union had ever happened, when the head was completely separated from the capsular ligament, and when it did not communicate with the rest of the body by the round ligament.

"When any one inspects the specimens shown in the Hotel-Dieu, he must be convinced of the consolidation. For myself, I am firmly convinced of it, notwithstanding the contrary opinions of the English surgeons.

"Let us now consider the theoretical and practical reasons in favor and against the possibility of consolidation of intra-capsular fracture of the neck of the femur.

"It is said that the superior fragment contains very few vessels, that it forms a true foreign body in the articulation. This assertion is untrue; The head of the femur receives evidently vessels from the cotyloid cavity by means of the round ligament. These vessels, without being very numerous or very large, can nevertheless suffice for the nutrition of the superior fragment. Again: the synovial membrane surrounds the cartilage, and forming at its base a little *cul-de-sac*, which possesses distinct portions of cellular tissue, and in which will be found a number of vessels. As to the inferior fragment, it receives a great number by the principal nutrient artery of the bone, which enters at its posterior part, and which forms in the whole extent of the bone numerous ramifications which nourish it. Finally, by the arteries which surround it, by those which appear in the dignital cavity of the great trochanter, and by all those which penetrate in the spongy tissue of the bone, and which sometimes run over its surface. The fibrous tissue which envelopes the neck of the femur, contains a great quantity.

"It is therefore evident that the inferior fragment receives many more vessels than the superior one, whose vitality is less active, more languid; and that for effecting the consolidation, the inferior fragment which enjoys a free exercise of all these vital properties, performs almost alone the consolidation; but it is not less true, that the superior fragment contributes to the process. The assertion relative to the absence or paucity of the vessels destined to nourish the fragments, is therefore valueless; the anatomical examination of the parts completely refutes it.

"A cause which has been considered almost insurmountable to the re-union of the fragments of the neck of the femur, is the absence of periosteum about this part of the bone. Here is a grand error; the neck of the femur possesses a periosteum, thin, no doubt, but still apparent, and if not so capable of nourishing as that of other bones, nevertheless it is real. This thinness is a difficulty, and by no means an insurmountable obstacle to consolidation.

"Others have said that the synovia continually bathes the fragments, and renders consolidation impossible. This reason would be good if the same anatomical disposition which is seen in various points of the osseous system prevented in any way the formation of callus. For no one is ignorant that fractures which penetrate into the joints consolidate very well, and the same thing happens to the olecranon and patella; in these places it cannot be contested but the synovia bathes the fragments. Every one admits the consolidation of the olecranon, but some contest that of the patella. I shall relate a fact, which proves this beyond all doubt. A man fell upon both his knees, and fractured his patellæ, one longitudinally and the other traversely; the femur was cured without any deformity; the latter separated for some lines. The man died some years afterwards, when the longitudinal fracture could scarcely be discovered, except by a slight inequality in the ridge of the fracture. The other was all consolidated. These cases prove that fragments, bathed with synovia, may be perfectly consolidated. It is not the presence of synovia that offers an obstacle to that of the neck of the femur. The true cause which impedes, or renders very difficult, the exact and solid consolidation, and above all the great deformity, is the displacement of the fragments, their want of apposition, whether the fracture is within or without the capsular ligament."

6. *Furious Delirium, consequent on the Repercussion of Erysipelas, cured by recalling the Inflammation.*—A man, aged forty-five years, was wounded the 24th November last, in the thigh, by a stabbing instrument, which penetrated four inches, and grazed the femoral artery without injuring it. M. Blandin found him in the following state: face red, pulse 100, head-ache; the edges of the wound red and painful; he was bled and put on low diet; the next day but one his state was very alarming; face red, pulse 130, look wild and stern; complete loss of intellectual faculties, furious delirium, violent movements of the limbs, &c. On interrogating his parents as to what had passed, M. B. learned that the thigh, in the situation of the wound, had become of a purple red some hours after his visit, with great heat and pain, and that they had applied on the part compresses dipped in cold water and vinegar; that under the influence of this treatment, the redness, and even the pain, had completely disappeared; and there remained but a slight yellowness in the part, and finally, that the cerebral symptoms came on suddenly afterwards with extreme violence. M. Blandin immediately bled him, applied twelve leeches behind the ears, sinapisms to the feet, a purgative injection, and friction, with tartar emetic ointment to the part which had been the seat of the erysipelas. The fifth day the inflammation returned, and extended over the internal and superior third of the thigh. The cerebral symptoms disappeared, and the patient complained only of head-ache and lassitude. The following days the erysipelas extended, and considerable fever set in; This was combatted by frictions with mercurial ointment, and in eight days the patient was completely recovered.—*Archives Generales.*

7. *Lunar Caustic Blister.*—In the 5th Vol. of the *Transactions of the Medical and Physical Society of Calcutta*, we find an article by J. C. Boswell, Esq. in which the lunar caustic is recommended as a substitute to cantharides for producing vesication. Mr. B. has used that remedy in pneumonia, phthisis, rheumatism, dysentary, &c. with, he says, decided utility. The blister is formed by slightly wetting the part, and then slowly drawing the stick of lunar caustic over the surface to be blistered, first longitudinally, and then across. The fluid is discharged by small punctures, in about ten hours after the application of the caustic; and no covering being used throughout, the surface becomes in two or three days sufficiently dry to admit of a second application, in the same place if necessary. Mr. B. has put on more than thirty of these blisters in the course of treating a pulmonary case, making the intervals longer as the cure proceeds.

The advantages which the nitrate of silver, applied as a blister, possesses over cantharides, according Mr. B. appears to be that its action is more immediate, and effect more powerful; it does not affect the urinary organs—requires no dressing, is easy of application, and always available—being so easily carried about.

Mr. Twining, the intelligent secretary of the society has appended to this paper some remarks confirmatory of the statements of Mr. Boswell. He says that he has found the caustic blisters very useful in chronic rheumatism of long duration, affecting the joints, and unattended with pyrexia, and with little or no acute local inflammation.

8. *New Method of operating in Ectropion.* By Dr. Dieffenbach.—The instruments necessary for this operation, are a small straight scalpel, with a narrow one-edged blade, a double-edged curved scalpel, a pair of forceps, and insect needles.

The operation, (we are detailing that for the lower eye-lid,) is commenced by a semilunar cutaneous incision, which is made at some lines distance above the inferior edge of the orbit, directing the instrument from right to left, so that for the right eye, the incision is commenced below the external; for the left, below the internal angle of the eye-lids, supposing that the operation is performed with the right hand. This incision, which must be parallel with the inferior edge of the orbit, should be about two-thirds of the length of the eye-lid, and exactly in the middle. When the incision has extended to the cellular tissue, the skin is to be detached for a certain extent from the tarsus, and the conjunctiva is then to be pierced, and the internal wound enlarged to the same size as the external. The conjunctiva and the tarsus which adheres to it, are then to be drawn through the external opening, a small portion of the conjunctiva being removed, the lips of the external opening are to be brought together, having between them the conjunctiva and the tarsal cartilage. This is to be effected by the finest insect needles, and threads twisted, as in the operation of hare-lip, after which the ends of the needles are to be bent, and cut close on the threads. The operation is the same for the upper eye-lid.

Cold applications are made use of, and if necessary the antiphlogistic treatment. The needles may be removed between the third and sixth day. The method of operating is explained by plates.—*London Medical and Surgical Journal, August,* 1832, *from Rust's Magazine.*

9. *Operation of Lithotomy for the Extraction of a Portion of Catheter from the Bladder.*—A stout athletic looking man who had lost one leg at Waterloo was brought into the operating theatre, at St. George's Hospital, in order to have a portion of gum catheter extracted from his bladder, by the ordinary lateral operation of lithotomy.

It appeared from the man's statement, that many years ago he had been in the Hospital under Sir Edward Home, with stricture of the urethra. For eight years after leaving the hospital, he had continued tolerably well, but subsequently to that period, he had been subject to occasional attacks of retention of urine, for which he was in the habit of introducing for himself a gum catheter of moderate dimensions. The catheter last in use was so antique that he had been obliged to cut off the rounded extremity, in consequence of flaws and cracks at the eye. On the 1st of the present month, he had the misfortune to let the instrument slip entirely into the urethra. After some ineffectual attempts to remove it by his own efforts, he went to a medical gentleman in the Vauxhall-road, who seized the catheter with a pair of forceps but could not withdraw it; it broke in the urethra, a large portion remaining in the bladder and vesical end of the canal. The patient describes the piece of catheter as forming a sort of projection in the perineum, and no doubt it might have been cut down on in that situation and extracted, if it could not have been removed by other means. The patient went his way and sought no further advice till the 3rd instant, when he repaired to another surgeon at Camberwell, who seems to have handled him rather roughly. This gentleman, whose incredulity appears to have been fully equal to that of the Lazarites, told the man he was a "bloody rogue," and summoned the new police. This sort of gen'darmerie surgery did not satisfy the patient, who went to another doctor, and another, and ended at last by coming to the hospital where he had formerly received benefit. This was on the 4th inst., but the operation was not performed until the 8th. In the interim he suffered much. He had constant desire to make water; passed little at a time, and that with pain; had violent pain in the right lumbar region, but not in the glans penis; had alkaline urine with deposition of mucus, and as we believe, the phosphates; and although some narcotics were administered, he spent but a miserable time of it.

On the 8th, as we have stated, the operation was performed by Mr. Brodie. It was the usual lateral operation, but the incisions were rather smaller than are made for the extraction of a calculus. Mr. Brodie employed his beaked knife, and the prostate was divided, the forceps introduced, and the gum-catheter extracted with Mr. Brodie's general precision, facility and despatch. The catheter was moderately curved, five or six inches in length, cracked in several places, and incrusted with a deposition of the phosphate of lime. The patient bore the operation without a murmur, and on the 15th, when we saw him, the wound was nearly healed, and the urine almost natural. Mr. Brodie made some valuable remarks upon the case to the gentlemen assembled to witness the operation. He said that he had never seen a similar accident occur before, although he had seen several instances of other foreign bodies slipping into the bladder, and forming the nucleus of stone. One of these cases was rather curious. A gardener laboring under stricture, conceived the ingenious but unsafe idea of employing the bougies which nature placed in his way. When laboring under more than ordinary difficulty in making water, he introduced a flower-stalk into the urethra and dilated the passage. But flower-stalks are brittle, and unfortunately one broke at last in the urethra, and a portion slipping into the bladder formed the nucleus of a stone which was afterwards removed by operation. Mr. Brodie pointed out the mortar-like incrustation of the catheter which he had just extracted. Now I know, said he, without resorting to any chemical analysis, that this incrustation is the phosphate of lime, because in such cases it always is so. The foreign body in the bladder becomes a source of irritation and inflammation—the inflamed mucous membrane of the bladder secretes mucus—and from that mucus is deposited the phosphate of lime. It is true that if the case went on other depositions would occur. The irritation, which had its primary seat in the bladder, would be propagated to the kidney, to the system. The *secretion of urine* would then become depraved, and the triple phosphate would be formed in the kidney, and this mingling with the phosphate of lime in the bladder, would form the fusible calculus.

Mr. Brodie explained why he had not attempted to remove the catheter by Sir Astley Cooper's urethra forceps, or by a pair of his own, which he shewed, and which exhibits several improvements on the former. He considered that the foreign body had been in the bladder for some time, that it was described as being of considerable size, that he might have failed in its extraction, and would have irritated further a part much irritated already. These and other considerations determined Mr. Brodie to cut into the bladder without any previous attempts with forceps, and the event has confirmed his judgment.—*Medico-Chirurg. Review.*

10. *Native Lithotomists in India.*—The Celsian operation, "by the gripe," would seem to be the rudiment of a scientific and successful lithotomy. In India the native operators employ it, and were we to credit the stories they tell us, with very great success. Our experience, however, is sufficient to prevent our being deluded by such narrations. Mr. King, of Patna, gives an interesting account of a native operator and his operation.—*ib.*

"At Poonerai, a village 3 coss from Patna, resides an Hujam of the name of Chukkun; who says that he has performed the operatien of lithotomy on 35 persons, one only of whom died: in that case the stone was of a very large size, and broke into several pieces; the patient died on the 3d day after the operation. He shews 20 calculi, some of which are of considerable bulk. The instrument he uses is something like a clumsy made pen-knife, and he describes his mode of operating as follows.

The patient being put in a convenient poisture, and held firmly by several persons, an assistant presses the lower part of the abdomen, in the direction of the pelvis. The operator introduces his fore and middle fingers into the rectum, and crooking them so as to obtain a hold of the stone, he brings it towards the perinæum, till it becomes prominent there. He then makes an incision over the stone, (on one side of the raphe, and about an inch from the anus,) and extracts it. The urine passes through the wound for some days and the cure is generally completed in from twelve to twenty days. Chukkun acquired his art from his father, and has now taught his son to perform the operation. As this man keep no register of the cases, his statement may be liable to errors, respecting the number of persons on whom he has operated; and probably a liberal deduction should be made from his account of success, which it is his interest to magnify. In fact, it is almost an invariable custom with native operators, to assert that their operations are attended with certain success; and that cures in their hands are infallible. At the same time we must acknowledge, that they evince much cunning and dexterity in assigning reasons for not operating on unfavorable cases. They operate with a view to *cure*, and would not hazard their reputation for the prospect of alleviating continued suffering, when they did not expect ultimate recovery."

11. *Cases of Phlebitis—Advantages of Free Openings.* Case 1. —*Phlebitis after Venesection—Operation—Recovery.*—Ensign H. æt. 19, resident in India one year, was bled to syncope (lbijss) in the night of Feb. 14, 1827, for dysenteric symptoms. He used the arm much in the night, and the bandage came off. The night of the 15th was very restless, he vomited frequently, had much heat of skin and head-ache, with furred tongue; pulse 120. The arm was painful, but not swelled, there was a little redness round the puncture, and on pressing around it with a sponge, a small quantity of pus and blood was discharged. He had taken since the 14th a scruple of calomel and two of blue pill. *Wound closed with sticking plaster—cold lotion —and purgative medicine.* At 2 P. M. the arm was more painful—skin hot and dry—had vomited—more pus pressed from puncture. In the evening eighteen leeches were applied, and afterwards a compress was put on four inches above the puncture. *Calomel and opium—salines with laudanum aud antimony—bath.* On the 17th the arm was more swollen below the compress. There was little alteration till noon of the 18th, when there was increased heat of skin, the arm was more painful and swollen towards the compress, and more matter was pressed from the wound by drawing the finger down the vein, from the compress to the puncture. At 3 P. M. the vein was swelled up to the shoulder, with great constitutional irritation. The following operation was performed.

"An incision was made with a scalpel through the integuments, and the median basilic vein exposed, a director was then introduced through the puncture, and with a probe pointed bistoury the opening was extended above and below as far as there was any appearance of pus, which was carefully extracted by pressure, and the wound closed in the usual manner. The application of the compress was continued till the 22d in the morning.

About oz ij. or oz.iij. of pus were evacuated, and the wound was dressed with a resinoterebinthinate ointment. In the course of half an hour the constitutional irritation had remarkably subsided, and the countenance, previously extremely anxious, was more composed. The same plan of treatment was persevered in, the patient taking a calomel and opium pill at night, with a diaphoretic opiate and aperients. On the 19th he was ordered quina, arrow-root with wine, and camphor. On the 20th, he was improving, the arm was dressed and better: it discharged freely. In the evening he was worse, the pulse being much quickened, with pain in the chest. *Calomel and opium and an anodyne.* After the increase of heat perspiration occurred, and in point of fact there was a paroxysm of fever, though without any perceptible rigor. Next day he was better, but had a slight cough. We need not pursue the details. On the 24th the arm was well, and some derangement of the hepatic function with a slight cough, was his sole complaint. He soon perfectly recovered.

Case 2.—*Phlebitis after Venesection—-Operation—-Recovery.*—John Palmer, private, æt. 24, resident in India two months, was admitted, March 16th, 1827, with acute hepatitis, for which lbij. of blood were taken from the right cephalic vein. The treatment for inflammation of the liver was continued; but on the evening of the 18th, he complained of pain in the arm, which was swollen, and the vein was found inflamed—pulse 100—head-ache. On the next morning he was free from fever, but the arm was as before. A compress and bandage placed on the vein, above the puncture, and the whole arm laid on a soft poultice. In the evening, the arm was more painful and swollen, the inflammation reaching as high as the compress, though no pus appeared at the puncture—pulse 100—head-ache—fever—anxious countenance. *Calomel c. Pulv. antim. et Op—Baln.* He passed a feverish night, but next day he was cool, with furred tongue and anxious countenance; a little pus could be pressed from the wound.

"An incision extending from one inch below, to one inch above the puncture, was made into the vein; its coats were found to be much thickened, its cavity larger than natural (on account of the compress placed on it above), and containing from about two to three drachms of pus. The inflammation does not extend beyond the compress; but a little pus was pressed from a portion of the vein next the hand." The wound was dressed with ungt. resin. flav. with a little oil of turpentine added to it. the arm again placed in a poultice."

He was ordered quinine, and under its exhibition, with occasional purgatives, no further unfavorable symptoms occurred. On the 23d, he is reported as getting rapidly well and in a few days the arm healed.

Case 3.—*Phlebitis after Venesection, fatal—Pus in the deeper Veins.*—T. H. private, æt. 22, in India five months, admitted June 27th, 1827, with feverishness and headache. The median basilic vein was opened, but, not bleeding freely, the cephalic in the same arm was cut, and lbj. of blood extracted, with relief. He was leeched thrice, and appeared to be doing well, till the morning of the 30th, when the arm was painful; both punctures were inflamed, but the inflammation appeared to extend up the cephalic vein, and not up the basilic. A pad and bandage were applied about the centre of the arm, and cold lotion used. In the evening the arm was worse, with fever. The cephalic vein was laid open as in the former cases, and oz.ij. of pus discharged from it, a little being pressed out of the median basilic, and a little from the puncture in the basilic. The arm was laid in a poultice; the treatment much as in the former cases. On July 1st, he seemed better, a little matter oozed out at the puncture in the median basilic, without apparent inflammation; the gums were tender. On the 3d, we find no pain or inflammation in the arm, the wound suppurating and looking well. Ordered quinine, and wine in arrow-root. On the 5th he had pain across the pit of the stomach, and leeches were applied. In the evening, he had that all but fatal sympton in such cases, severe rigors and fever. The next day he looked ill, his appearance indicated mischief, though the arm showed nothing amiss. The median basilic vein was laid open, and found to have a black appearance. In the evening of 7th, he had another attach of fever; and on the 8th, he said he felt better, "though he did not look so," a very characteristic symptom. He gradually sank, and died at 7, P. M. of the 11th.

On dissection, the veins which had been operated upon, looked healthy, but the deep-seated veins, especially the brachial, were found full of pus. No mention is made of the state of the liver or lungs.

We have analysed these cases, because we think they inculcate an important point of practice, that of laying open freely an inflamed and suppurating vein. The secondary consequences of phlebitis, as of other injuries, are more frequently observed when matter is locked up, or does not freely escape. It is on this account that diffuse inflammation of the cellular membrane, between muscles, is so much more frequently followed by visceral deposites, than diffuse inflammation of the subcutaneous cellular tissue. We do not place much confidence on the employment of pressure by a pad; on the contrary we are tempted to believe that it is injurious, by the mechanical obstruction offered to the circulation of the limb. Every surgeon is aware how much any ligature, or compression on an inflamed limb, interferes with the natural processes of reparation. We suspect, from the manner in which the foregoing cases are drawn up, that the reporters are not aware of all that has been done in the investigation of the secondary inflammations. Thus in the last case, we are informed, after the occurrence of rigors, that "the arm does not seem to be at all connected with his ill-health, but the disease to be in the liver, in the bowels." No surgeon who had seen many cases of the purulent depots would make such a remark. However, we again beg to draw particular attention to the practice employed in some of these cases, and to advocate most warmly the free opening of inflamed veins, when the surgeon has reason to believe they contain pus, which does not escape with the utmost freedom and facility.—*ib.*

12. *Treatment of Croup.*—[The following is the original statement which we published three years ago on this interesting subject. It first appeared in the London Lancet, and is from the pen of Mr. Surgeon Kemble, of Knowle, in Warwickshire. The success of this gentleman is represented to have been great.]—*Boston Med. and Sur. Journal.*

In this district, the *croup*, from local causes, is unusually prevalent; and it has fallen to my lot, partly from the success of my plan of treatment, to witness more specimens than commonly occur to one person. We have also, at times, abundance of *bastard croup.* It is unnecessary here to dwell upon the symptoms, which, under the name of the former, do, with the ordinary treatment, so often lead to a fatal termination; but there can be no doubt that, if activity of "antiphlogistic practice" and prompt attention only were requisite, the results would be far otherwise than they are reported to be, as there are very few infantile maladies to the rapidity and danger of which the public and the medical profession are more sensibly alive. I have been induced to think that the fatality in croup is mainly attributable to an erroneous pathology, and, consequently, to the misdirection of our attentions in the mode of treatment; and death appears to me to be produced, at least in the generality of instances, not by the systematic violence of the peculiar pellicular inflammation, nor by the often trifling quantity of plastic effusion which attends it, but to be directly owing to the spasm which is obviously present, and operative, at least to a certain extent, in every case. That the actual straitening of the oreal aperture by false membrane is not generally the cause of death, there cannot be much doubt. I have never witnessed an examination after death by croup, where an opening has been left, such as to lead those present to think it adequate to the further prolongation of life; and in the recorded cases of cynanche laryngea in adults, this circumstance is still more forcible, while it is a strong concurrent fact, repeatedly observed, that the fatality in croup is in no wise proportioned to the extent of the tube affected, but rather correspondent to its site; those cases being most grievous, rapid, and fatal, in which the inflammatory process is developed directly upon the apparatus for contraction. Again, that inflammation in an open passage, lined by mucous membrane, and occasionally so limited as to leave but slight traces after death, should proceed rapidly to a fatal termination, by its effect on the system, is unsupported by analogy, and would be a very remarkable occurrence in the history of disease. I am therefore led to conclude, that the peculiar complex condition which we denominate inflammation, is not, in croup, the principal cause of death.

To preclude the admission of noxious bodies, nature has endowed the entrance of the lungs with a degree of irritability, very exquisite, even in the healthiest state. A morbid increase, or exaltation of the natural irritability, accompanied with afflux (whether cause or consequence), and the symptoms arising from these two states, constitute inflammation. Morbid irritability, occurring in the muscular and musculo-ligamentous tissues, exhibits those phenomena of abnormal and irregular contraction, which we call spasm. Without canvassing their specific nature and difference, or the reciprocal power of each to produce the other in every case, it is evident that spasm is of very frequent occurrence in textures immediately subjacent to an inflamed organ, or associated with it in office. Whenever the mucous lining, or other texture near the extremity of an open passage, is inflamed, the muscles connected with it, and particularly those subservient to its closure, are sure to partake of the spasmodic condition. Inflammation of the urethra, inflammation of the neck of the bladder, and abscess in the vicinity of the rectum, are obvious examples; and the levator, the acceleratores, and the sphincters, are excited to frequent and irregular contractions. The natural and morbid irritability of parts are, I believe, pretty generally, in a direct ratio to each other, exclusive of circumstances of situation.

In the part attacked by the croup, the natural aptitude to contract every moment, for the purposes of self-preservation, is much greater than in the rectum and urethra; the apparatus is more complicated; the function is vital. A brief interruption, in the other cases but of little moment, is here, by the non-expansion of the great pulmonary receptacle, an obstacle to the return from the head; from that cause, an increased portion of the ascending current, unable to penetrate the cranium, is diverted, by the superior laryngeal branches, to the parts before oppressed; and thus the reflected consequence of the contraction of the aperture of the glottis by a spasm, is to aggravate its primary cause—a specific inflammation of the mucous membrane; that secondary effect is productive of still further spasm, and, after repeated paroxysms, each depressing still lower the vital power, harassed by ineffectual cough, distressed for breath, and laboring at the heart, the little subject is destroyed. The immediate cause of death is a condition of the brain, which is inadequate to maintain the organic stimulation requisite for the continuance of those functions which constitute visible life; that state arises from non-oxygenation, the non-performance of which, in the very last act, is perhaps mainly to be referred to the presence of mucus, and in some degree, perhaps, to the peculiar effusion in the larynx and trachea.

From the preceding view it follows, that were it possible, by the maintenance of narcotization, by the free use of antispasmodics, or by their joint co-operation, to effect the removal of spasm, to prevent any vexation but that arising directly from the inflammatory process, its course would be rendered milder, and it would probably re-approach to the nature of the common catarrhal affection, with which it always appears to commence. Time would be gained to establish some control over the local action by the ordinary means; and, for the removal or consolidation of the lymph, nature might be freely trusted to her own resources. A trial of considerable magnitude has convinced me that this view is substantially correct. The supposition of the important influence of spasm, derives confirmation from the success of the practice, which would be otherwise unaccountable. I am of opinion, that all the worst symptoms of the malady are attributable to the *spasm only;* that there is not any thing in the specific nature of the action present, nor in the parts affected (excepting their great readiness to take on spasm), which should *necessarily* produce a very heavy mortality; and I feel satisfied that if, instead of combating inflammation, we resolutely, and from the commencement, address ourselves to subdue spasm, the termination of the great majority of the cases of croup will be far other than it has been. At all events, I can state distinctly, that in my hands the subjoined plan has been so remarkably fortunate, that I have scarcely seen a fatal case since it has been adopted; and it has been equally successful in the hands of other persons at a distance, who have been apprised of these facts. It possesses the rare advantage of making *no inroads upon the patients strength;* for I have frequently seen a child play, and, to all appearance, as well as ever, *on the third day*, after having had all the symptoms of true croup. And it may well be demanded, Of how few children could that be said, if they were merely subjected to the ordinary treatment *without any malady*? Bleeding "freely" with leeches, and perhaps from the arm, blistering the surface of the neck, applying caustic to the fauces, drastic purging, calomel by cart-loads, and antimony "*usque ad nauseam*," are quite enough to exhaust the life of an irritable and delicate infant. I never bleed or blister a child in croup; I have never thought it requisite to do so, since I have adopted the plan alluded to, although such an auxiliary practice would be in no other respect incompatible, than as tending to invalidate the general strength. The treatment I allude to consists in confining the child to a uniform and rather warm temperature, giving an emetic of ipecacuanha, and, in an hour after, commencing the following mixture:—

℞. Rad. Valerian. Pulv. ʒij.

Oxymel Scillæ ℥i.

Tinct. Opii gtt. xx.

Aq. Distil. ℥i. M.

I administer a teaspoonful every hour, if the child is from two to five years old: if from five to eight, every five-and-forty minutes, so as to maintain the anodyne effect of opium, and the sub-nauseating, expectorant, antispasmodic effects of the squill and valerian, until the symptoms are removed, which commonly happens in ten or twelve hours, and which I have never seen protracted beyond eight-and-forty. On their subsidence, I have, in general, given a brisk dose of calomel and jalap.

This plan will also be found exceedingly efficient in hooping cough; and I can state, that when it is uncomplicated with tubercular disease, I have found my method more certainly and more speedily of use than any of the numerous procedures which are usually recommended.

13. *Croup.—To the Editor of the Boston Medical and Surgical Journal.* Sir,—About thirty years ago, when my attention was first turned particularly to the subject, the croup was justly considered, according to the practice of that day, as one of the most incurable of all acute, febrile diseases. Probably not more than one patient in six recovered from the complaint. At the present time, though it is a malady that demands the earliest and closest attention, and consequently is still very dangerous, yet when circumstances are favorable for putting the most approved practice into complete operation, we rarely lose more than one case out of eight or ten. Our dependence is now upon *acrid, deobstruent emetics, calomel* and *opium.*

When called to a child laboring under croup, I would instantly give from half a scruple to a scruple of calomel, to be immediately followed by an emetic of the following mixture:

℞. Tinct. Sanguinar. Canad.

Syrup. Scill. Marit. āā ℥j.

Decoct. Polygal. Seneg. ℥ij.

Pulv. Ipecacuan.

Pulv. Sulph. Zinc. āā ℈ij. M.

Half an ounce of this mixture is to be administered *perseveringly*, every five minutes, till *free* vomiting ensues. Instead of the calomel which I previously give, some eminent practitioners add three or four grains of turbeth mineral, as an efficient deobstruent, to the first dose of the acrid emetic mixture. The preparation, also admits of a considerable variation, as respects the proportions of blood-root, squill, and senega.

In ordinary cases, free vomiting always produces speedy, though often only transient, relief. As soon as this occurs, from three to five grains of calomel, combined with three to five minims of laudanum, or a sixth to a fourth of a grain of opium, are administered every hour or every two hours, till the force of the disease yields, or catharsis makes it necessary to lessen the quantity of calomel, or to suspend it altogether. Small doses of opium are necessary, till the recovery is established.

Every time that a paroxysm of difficult respiration and distress returns, the acrid emetic is again repeated, as at first. Indeed, it is generally necessary to repeat the emetic, occasionally, upon any unpleasant symptom supervening, till there is ejected a certain kind of *glairy mucus*, which, from its consistence, falls into the vessel, in a manner somewhat resembling melted lead or melted tin.

It is also well to give regularly, every hour, as much tincture of sanguinaria, or decoction of senega, as can well be borne without vomiting. Unless the disease is broken up by the first emetic, which is sometimes the case, it is indispensable to this method of practice that the patient be kept *uniformly* under the *antirritant* effect of opium.

I have often known mild cases to yield easily to calomel and opium, without any emetic; and they are frequently managed with sanguinaria, in decoction or tincture, or with senega, without calomel. But *opium, given regularly, so that a second dose is taken before the effect of the first is much diminished,* is indispensable in either case, if we would expect to arrive at any thing like certainty in the treatment.

It is perhaps needless to remark, that we avail ourselves, according to circumstances, of the warm or hot bath, fomentations, liniments, sinapisms, epispatics, &c.; in a word, of all the common adjuvants.

The outline of this treatment is to be found in Professor Tully's Essay on Sanguinaria Canadensis (American Medical Recorder, Vol. 13, Jan. 1828). It has been successfully practised by the most eminent physicians in this State, between twenty and thirty years; so that in this section of the country, croup, among the physicians who know how to treat it, is nearly as much divested of its terrors as is the case with the small-pox. We are enabled entirely to dispense with bleeding; and we consider antimonials, in every shape, as among the most doubtful, not to say exceptionable remedies. True, they sometimes succeed; but when they fail, they are apt to be attended with a peculiar irritation, which is generally as unmanageable as the original disease.

In my own practice, I adopted this treatment, in substance, in the year 1810, with very decided success, at a time when the croup was so prevalent that it amounted to a kind of epidemic.

I have but one further remark. In no disease are greater resolution, decision, and firmness, demanded. If the physician has the weakness to be more afraid of his remedies, particularly of opium, than he is of the disease, he can never be expected to treat this malady with success, but will be ever liable to lose a majority of his patients.

*Connecticut, February,* 1833. SENEX.

14. *Affections of the Head produced by Quinine.*—Quinine was found to produce some remarkable affection of the head, in almost every case in which it was administered at Jubbulpore, in 1829. In one officer it caused transient deafness; in another vertigo; in a third intolerance of light, to such degree that the medical men were alarmed least effusion on the brain should take place. A fourth European was subject for a short time, to much confusion of ideas, and all sorts of chimeras after taking Quinine. A Portuguese was affected with tinnitus aurium, and deafness. In all these cases, the quinine acted favorably on the fever, for which it was administered. In the same note favorable mention is made of the efficacy of Rohena Bark (Swietina Febrifuga) in fever cases; but large doses of that remedy were also found to produce vertigo.—*Calcutta Transactions, Vol. V.*

15. *Spinal Apoplexy, or Extravasation of Blood in the Spinal Canal.*—Case 1.—A child aged 7 days 1st September, 1818, was observed not to suck, and appeared as if he were prevented by something which impeded the motion of his tongue. Through the following day he cried frequently, and still did not suck; in the evening he was seen by Mr. White, who found the jaw clenched by spasm, but by very little force it could be opened. On the third day he was seized with convulsion, which recurred at various intervals, sometimes in the form of tonic spasm of the whole body, and sometimes of violent convulsive agitation. On the fourth the convulsion continued, and he died in the afternoon.

*Inspection.*—No disease could be detected in the brain. In the spinal canal there was found a long and very firm coagulum of blood lying between the bones and the membranes of the cord on the posterior part, and extending the whole length of the cervical portion.

This is the only case that has occurred to me of this remarkable affection; but, as it appears to be uncommon, and to present some very interesting phenomena, I am induced to add the following examples.

1. A lady aged 40, had headach and pain of the back; after a few days the pain of the back became very acute, and violent convulsion took place, which was fatal, after continuing five or six hours. All was sound in the brain, but extensive extravasation of blood was found in the spinal canal, which was most abundant about the seat of the pain.*

2. A gentleman, aged 61, had just arrived in Paris, from a long journey, when he complained of pain of his back, extending from the cervical vertebræ quite to the sacrum. After a few hours he was seized with paraplegia, and incontinence of urine and fæces; and he died while the physician was talking to him, who had been sent for on the occurrence of the palsy. There was extensive extravasation of blood in the spinal canal, under the membranes of the cord. At the lower part it formed a mass like a bouillie of bullock's blood, in which the substance of the cord could not be distinguished, as far as the third dorsal vertebra; and above this, where the cord was entire, it was of a deep red color, and very soft.†

3. A young lady aged 14, had headach, and pain in the back, with a tendency to sickness on sitting up. At the end of the week there was a sudden and violent aggravation of this pain, followed by general convulsions, which were fatal in five or six hours. The spinal canal was found filled with extravasated blood, in the lumbar region, which had been the seat of the pain. The brain and all the other viscera were sound.‡

4. A miller, in lifting a heavy sack suddenly, lost the use of his lower extremities, and died in fifteen days. Extravasated blood was found, mixed with sanious matter, in the spinal canal. The mem-

* Ollivier *ut supra.*

† M. Gaultier de Claubry.

‡ Chevalier Med. Chir. Trans. vol. iii.

branes were inflamed, and the nerves of the corda equina appeared rotten, as if they had been long macerated in putrid water*

5. A gentleman died of a disease which was considered as apoplectic, but in which he retained his mental faculties to the last. No disease was discovered in the brain, but there was a great quantity of extravasated blood in the spinal canal.†

6. A man received a violent blow on the three inferior lumbar vertebræ, by a log of wood which fell upon him; he died in four hours. Extravasated blood was found in the spinal canal, but the vertebræ were entire, and the cord was healthy.‡

7. A boy aged 14, received a violent jerk of his neck by a cord which was thrown over his head as he was swinging forward in a swing. He felt no bad effect at the time, but, after some time, became inactive and weak in the limbs, with stiffness of the neck, and difficulty in moving his head. Nine months after the accident the weakness of his limbs increased to paraplegia; and soon after he had paralysis of the arms, with retention of urine. He had been a short time in this state when he was seized with violent pain in the spine; he then had difficult and quick breathing, which was first observed during sleep, but afterwards continued while he was awake; and he died after suffering severely from it for two days. His death happened about ten months from the injury, and a few days after the violent attack of pain in the spine. A large quantity of extravasated blood was found in the spinal canal, betwixt the bone and the theca vertebralis. It was partly coagulated and partly fluid, and appeared to have come from the upper part of the canal about the second or third cervical vertebra.§

16. *Destruction of a Portion of the Spinal Cord..*—A man, whose case is related by Mr. Copeland, had paraplegia, dysuria, obstinacy of the bowels, and a feeling of tightness across his belly, as if a broad band had been bound tightly round it. His health had been declining for more than a year, and the commencement of his complaints was ascribed to having violently sprained his back in lifting a heavy weight. After being confined to bed with perfect paraplegia for three months, he died of gangrene of the nates. On dissection, no disease could be discovered in the vertebræ. Within the last dorsal and first lumbar vertebræ. The spinal cord was entirely wanting for more than two inches. The membranes which there formed an empty bag, were unusually vascular and much thickened.‖ On the other hand, Ollivier found four inches of the cord entirely wanting in a child aged eight years, who died of extreme marasmus, with caries of the vertebræ, but without loss either of sensibility or motion of the limbs. Velpeau has described several cases in which, in connexion

* Chevalier, *ut supra.*

† Du Hamel.

‡ Morgagni. Epis. 54.

§ Howship's Observations in Surgery and Morbid Anatomy.

‖ Copeland on Diseases of the Spine.

with caries of the vertebræ, the cord was completely destroyed for the space of several inches, the patient having died of gradual marasmus without any appearance of paralysis; and in Majendie's Journal, a case is described, in which the cord had become quite liquid, through two-thirds of the dorsal region, and one-third of the cervical. The arms were paralytic without loss of sensibility, but the legs were not affected. Ollivier has also observed in two cases a remarkable wasting or diminution of size of the cord. The one was in an old man without any particular symptoms; the other in an idiot, with permanent contraction and wasting of the limbs.—*Abercrombie on Diseases of the Brain and Spinal Cord.*

17. *Case of Aphonia, treated by Nitras Argenti.*—A young woman had gradually lost her voice, from repeated attacks of catarrh; her general health was quite good. During deglutition, she experinced an uneasiness in the larynx. When she did not make any very strong effort to speak out, her voice resembled a low whisper; but if animated and anxious to exert her speech, a noise, like a succession of shrill whistles, or of the mewings of a kitten was produced. M. Trosseau treated the case by the local application of strong solution of lunar caustic introduced by means of a sponge affixed to a bougie, and gently pressed over the opening of the larynx. The operation was followed by retchings, vomiting, and a cough which nothing could allay. These distressing symptoms abated in about a quarter of an hour, and then the patient assured M. T. that she felt little pain when the caustic was applied; and she expressed her astonishment that already her voice was much stronger, and that she could speak louder than she had been able to do for eight months. She was ordered to gargle her mouth and throat with a solution of alum. The retching and vomiting recurred at intervals till the following day, and the pain of the operation continued for a day or two longer. The patient was, however, unable to drink, and to swallow soup, &c. What is strange is, that although almost immediately after the application of the caustic, the voice was so much improved, at the end of ten days the aphonia was as bad as ever. The remedy was re-applied, but in a still stronger form. A sponge, dipped in a saturated solution of the caustic, was introduced as far as the opening of the larynx, and about ten drops allowed to distil into it. Immediately a violent convulsive cough arose, by which most of it was expelled in the Doctor's face! The pharynx, tongue, and lining membrane of the cheeks were rendered as white as milk. Vomiting and a severe cough came on; fortunately there was but little pain, no fever or constitutional disturbance. Her voice did not receive the speedy amendment, as after the first operation. The cough was very troublesome for twelve days; but the voice appeared to have obtained strength, although it was very hoarse. after the 13th day, she was unable to speak for some minutes in succession, and the aphonia never afterwards returned. The voice at first hoarse and occasionally squeaking, became more and more clear; however, after a long walk, or exposure to cold, or too much talking, the hoarseness still returns, but not the aphonia.—*Journ. Univers. et Hebdom.*

18. *Case of Rheumatism, treated by the Local Use of the Acetate of Morphia.*—A woman aged 28, had a smart attack of the rheumatism in both knees, the shoulders, and wrists; the pains were constant, and the affected parts were considerably swollen and red. She was freely bled from the arm, without relief—blood sizy. A blister on each knee ordered, and the raw surfaces to be dressed with half a grain of acetate of morphia; for the three subsequent days she was quite free from all pain—the quantity of the morphia increased to three-quarters of a grain. In two days more she had completely recovered.

*Case* 2. A man, aged 27, was seized with violent pain in both knees, on the 15th March. On the 22d, the joints were blistered, and the morphia applied in the dose of a quarter of a grain. In two days the pains were much relieved. On the 28th, he was pronounced cured.

*Case* 3. A young man, aged 16, suffered unremitting pains in the knees, wrists, and elbow-joints, which were swollen, red, and very tender on pressure, and incapable of being moved. For three nights he could not sleep, from the severe suffering; pulse full; skin burning hot; he was bled and starved. Next day the pulse was more favorable, but the pains rather worse: a blister to be applied to each knee, and the surfaces to be dressed with a quarter of a grain of the acetate of morphia. This treatment was continued for four days, when the patient made no complaints but from the blistered surfaces.—*ib.*

19. *Case of severe Sciatica cured by Bloodletting.*—A. R. aged 39, had suffered for a month, from excruciating pains in the upper and back part of the right thigh; they shot down on the outer side of the limb to the ankle and foot, and generally ceased with a distressing shaking or trembling of the extremity. These paroxysms returned almost every hour, and were so agonizing as to force the patient to scream out. While they lasted, the whole limb appeared to be affected with a tetanic spasm, the gastroc-nemii muscles being drawn into hard, knotty swellings. During the intermissions, there was a constant, slightly-hot pain along the course of the sciatic nerve. He was largely bled from the arm, and 30 leeches ordered over the sciatic notch, to be followed by opiate poultices. On the following day, the number of paroxysms were reduced by one-half, and the character of the pain changed from cramp and the feeling of burning, which shot like a rocket from the hip to the toes, to that of cold water gliding rapidly under the skin in the same direction; leeches to be re-applied. In two days the pains had quite disappeared; and in order to confirm the cure, the patient was ordered to rub in twenty drops of croton oil on the outside of the thigh; a copious eruption of pustules was produced, and, in six days more, the patient was discharged cured.—*ib.*

20. *Paralysis of the Portio Dura cured by Moxas.* A. M. aged 43. The mouth was much drawn to the right side, and the right commissure pulled considerably upwards, while the left was directed downwards, and inwards, leaving the front teeth exposed; the left cheek was much stretched, and closely applied to the alveolar arches; the left eye-lids closed imperfectly, and thus, part of the cornea was unprotected; the cause of this was chiefly attributable to the upper lid being drawn up by its levator muscle. Considerable conjunctivitis had ensued, but the vision did not seem affected. The food was always involuntarily carried to the right side of the mouth; the saliva and any drink trickled from the left angle of the mouth down his chin; the speech, at first, was not affected, but afterwards he could not pronounce certain words; the sensibility of the parts, and the functions of the taste and smell were perfect. The symptoms had existed for seven days, and no assignable cause could be stated. A moxa was applied over the condyle of the lower maxilla, and good effects were apparent, on the second day, in those parts which are supplied by the the middle or transverse branch of the facial nerve; a second moxa was, therefore, ordered over the angle of the jaw, and speedily the speech became more distinct, the deformity of the mouth less, the food was more easily and steadily retained between the teeth in mastication, and liquids could be better retained. When the patient attempted to pronounce the letter "O," the hiatus of the mouth was decidedly less on the affected side than hitherto; still the motions of the left eyelids were very imperfect, and, therefore, two days after the last moxa, a third was ordered over the course of the ascending twig of the nerve. The effects were most gratifying; for on the same evening, the patient could close the lids quite accurately, the ophthalmia disappeared, and in 17 days the patient was discharged well.

*Reflections.* This case shews satisfactorily that such affections of the facial nerve are frequently local, and not at all connected with the cerebral disease. The occurrence of the paralysis successively in the different branches of the nerve, and the effects of the treatment, first on one of these branches, and then on the other two, are very interesting, and confirm the opinion just stated. The propulsion of the food between the teeth and cheek of the right, or healthy side, causing it to be distended, and requiring the finger to bring the mouthful between the teeth, is rather contradictory to what is usually observed.—*Journ. Univers. et Hebdom.*

21. *Vitreous Humor Regenerated.*—A sergeant in the Garde Royale, received an injury on the left eye which burst the globe, and expelled the crystalline, vitreous, and aqueous humors. The palpebræ were ecchymosed; the conjunctiva red and bloated; and the lower part of the cornea was separated from its attachment to the sclerotic by an irregular cut, through which a portion of the iris protruded. The parts were washed, the iris carefully returned, the edges of the cornea approximated, the eyelids closed, and blood was drawn from the temporal artery. Ice to the head, sinapisms to the feet, constant darkness, and a vigorous diet were enjoined. During the night he was bled again, and next day cupping glasses were applied between the shoulders. It was supposed that vision would be forever lost, but *a notre tres grande et agreeable surprise*, the globe gradually refilled; on the 22d day, the cornea began to assume a healing aspect, and in about six weeks it was perfectly cicatrized. The natural form, and almost the ordinary volume of the organ were restored, and by the aid of a very convex glass, this soldier came to see objects distinctly, and continues to discharge all the duties of his office.—*Larrey's Clinique Chirurgicale—Glasg. Journ. Feb.* 1832.

22. *Cases of Vesico-Vaginal and Recto-Vaginal Fistula cured.*—Two cases of this dreadful deformity, in which operations were crowned with success, have been lately published. Such cases are always valuable, because they encourage surgeons to persevere in attempts at removing loathsome and almost unmanageable ills, when they might be utterly disheartened by failures by far too numerous.

*Case* 1.* Mrs. R. æt. 22, had labored under vesico-vaginal fistula since her first confinement, which had been protracted and severe; the finger passed readily through the fistula into the bladder, and for eight months many plans had been tried without success. M. Malagodi operated in the following manner.

Having placed the patient as if for lithotomy, he introduced the fore-finger of the right hand, covered with the finger of a glove, into the fistulous opening, and bending it, drew down the left border of the fistula to the orifice of the vagina; and then, with the other hand, he pared off the edges with a straight bistoury. The hand was changed, and the same operation repeated on the right border of the fistula. The pared edges were then brought in contact. To do this M. Malagodi re-introduced the right index finger into the opening, and brought down its left border, and with the left hand passed a small curved needle, fastened with a ligature, through it, near the posterior angle of the wound. A second and third needle were passed in the same manner, at equal distance from each other, and the opposite border was transfixed in the same manner, when the ligatures were tied, and the sides of the opening thus kept together. The patient was placed on her back in bed, a catheter kept in the bladder, and the urine allowed to flow constantly through it. Until the morning of the third day the water was passed through the catheter only, but then it began to escape by the wound into the vagina. On the fourth day it was found that the two posterior sutures remained firm; and on withdrawing them the union of the sides of the fistula was complete. The anterior ligature, on the other hand, had torn the left border of the wound, and nearly a third of the original fistula thus remained unhealed. The edges of this were regularly touched with the nitrate of silver, the

* Racoglitor Medico. Glasgow Journal, Feb.

catheter being kept constantly in the bladder. This plan was completely successful at the end of a few weeks.

*Case* 2* Isabella Pigot, æt. 28, was admitted into the London Hospital, Dec. 2, 1830, with vesico-vaginal fistula. The opening between the vagina and neck of the bladder was sufficiently large to admit the point of a fore-finger, and seemed to involve a small portion of the commencement of the urethra; it was oval, its long diameter lying transversely and about 1 3-4 inches from the orifice of the meatus urinarius. The flow of urine was constant, but the retentive power of the bladder was not wholly destroyed. The edges of the aperture were callous to only a slight extent. She stated that four months previouly she had been delivered of a still-born child after a protracted labor, in which the bladder had not been relieved. Her recovery was speedy. but since her confinement she had been unable to retain her urine.

On the 28th Dec. Mr. Luke proceeded to perform the following operation. The patient being placed as for lithotomy, a female catheter was introduced into the bladder through the meatus urinarius. Weiss's two-branched dilator of the female urethra was then passed per vaginam, and fully opened, by which the fistulous orifice was exposed. Attempts were made to pass tenacula through the orifice, but they were found too little curved, and two hooks were therefore employed, Their points were introduced about an eighth of an inch from the internal side of the opening, and brought out at the same distance on the external, when the sides were next pared by two semi-elliptical incisions made transversely in the vagina; to make the internal it was necessary to use a scalpel with one-half the blade bent at a right angle with the other. There was now some difficulty experienced in pressing the ligatures for closing the wound. Small curved needles held in a porte-aiguille would not answer. Mr. L. succeeded with larger ones less curved, passing one on each side of the urethra at a little distance from the edge of the wound; the ligatures when tightened perfectly closed the orifice. The dilator was withdrawn, and an elastic catheter secured in the urethra; the patient was then sent to bed, and ordered to lie on her face, and allow the urine to drain on a cloth.

No water passed by the wound till the 1st January, when the ligatures were found to have ulcerated from one side, and the edges of the wound to be so separated as to allow the escape of the whole of the urine. On the 3d the opening was larger than before the operation; the ligatures were removed, the catheter allowed to remain. 6th. Elastic catheter being stopped up was withdrawn. Another ligature was passed in the centre of the opening including more substance than before; the silver flat catheter was fastened in and the patient placed on her face. On the 11th the ligatures having produced ulceration were removed, but the wound still adhered; a clean catheter was introduced. 15th. catheter has become stopped; wound re-opened, and nearly of former size.

* Med. Gazette.

*March* 19*th.* Mr. L. pared the edges of the opening by carrying a single hook through the sides and excising them with a common scalpel; a single ligature kept them in contact. The patient was placed on her face and a catheter introduced as before. On the 26th, the ligature having produced ulceration, was removed; the sides of the opening were adherent, and all the urine flowed by the catheter, which was allowed to remain. On the 9th April the catheter was removed; the fistulous opening was perfectly closed. A stillicidium urinæ remained for a short time, but as the parts regained their tone it subsided. On the 25th she was discharged cured.

*Case* 3.* An Irish woman, æt. 30, was admitted into the London Hospital on the 24th May, under the care of Mr. Scott. A fistulous communication was found to exist between the rectum and vagina, and the fæces escaped through it into the latter. Her first labor had occurred about ten weeks previously, and during a severe pain whilst the child's head was in the vagina, the attending midwife passed the hand into the vagina, at which time the patient felt something give way.

The bowels having previously been freely opened Mr. Scott operated on the 13th June. He commenced by pulling down the vagina with a double hook, then pared off the cartilaginous edges with a probe-pointed bistoury, brought the raw edges together, and retained them in contact by means of three sutures. She was strictly confined to slops that the parts might not be disturbed by the occurrence of motions. During the night she passed her urine thrice and rested well. On the following night she was very unwell and fainted twice. On the 17th she was ordered some castor oil by the dresser, which was followed by a motion and the passage of a part of the fæces by the vagina. On the 26th Mr. Scott made an examination of the parts. The edges of the opening had united throughout nearly their whole extent, a small ununited portion only being left through which some feculent matter passes when she has a motion. This is the last report, and consequently the result of the operation is not yet determined.

There is one circumstance to be recollected in perusing such cases as the foregoing. The recto-vaginal, or vesico-vaginal fistula is usually the consequence of labor, and although a temporary cure be obtained by operations, the patients are liable to future pregnancies. We must not then consider the case as concluded when the female leaves the hospital cured. Future inquiries should determine whether that cure is permanent. Unfortunately such inquiries are too often neglected, or even when made, the issue, if unfortunate, is not published so eagerly as the former seeming success.—*Medico-Chir. Review.*

23. *Tic Douloureux of a Stump said to be cured by the removal of a portion of the Median Nerve.*†—There is one disadvantage attending hospital reports as at present managed. The reporter, whoever he

*Lancet, July 21, 1832.

†Medical Gazette, July 13, 1832.

may be, is usually in such a hurry to open his budget, that he will not stop to learn the issue of a case before he gazettes it as a cure. The consequence is that patients have been dead or dying, at the very period when the success of the treatment employed has been going the rounds of the medical journals. If the result is afterwards made known it is done so in parenthesis, and escapes many who read the former statement. We do not say the case we are about to notice is an example of this fault, and yet we suspect that the narrator is scarcely justified in setting it down at present as an instance of cure from a particular operation. We had false facts enough in medicine, before the modern system of hospital reporting opened a fresh vein of the poisonous ore.

*Case.* Eliza Burkett, æt 20, was admitted into the London Hospital, April 21, under the care of Mr. Luke.

"She stated that about three years and a half since, she was taken into St. Thomas's Hospital in consequence of severe injury to the left hand, occasioned by a fall. Every attempt to save it having been made, after two years, amputation became necessary, on account of her general health suffering considerably from the excessive suppuration and painful sensations in the arm; the stump did not heal favorably; she suffered distressing agony, and her health again became affected. She was discharged from the hospital, and afterwards admitted into cripplegate workhouse, under the care of Mr. Langstaff. At that time the surface of the stump presented an unfavorable appearance; the skin covering the ends of the radius and ulna was very thin, excessively vascular, and the ends of these bones seemed likely to cause its absorption. There was also a constant state of convulsive action of the muscles of the stump, accompanied with agonizing pain. Poulticing, opiate lotions, belladonna, and gentle pressure by bandage, were employed to lessen her sufferings, but without any good effect.

Mr. Langstaff afterwards performed amputation above the elbow-joint by the flap operation; and previously to securing the arteries he drew out each nerve to the extent of half an inch from the surface of the stump with a tenaculum, and cut through them to prevent their interrupting the process of cicatrization of the integumental parts. For some time she was relieved of all the painful sensations she had so long been distressed with; had no recurrence of hysteria or convulsion, and her health improved.

About two months after the last operation she applied at this hospital, complaining of severe pain in the stump, the extremity of which was inflamed and affected with spasmodic twitchings; she was constantly subject to headache, and was unable to obtain rest night or day; altogether her life was rendered so miserable that if relief could be procured, she was willing to undergo amputation at the shoulder-joint. The carbonate of iron, iodine, acetate of morphia, blisters in the axilla, leeches to the extremity of the stump, cupping at the nape of the neck, and various other remedies were employed, but without any beneficial effect, except the cupping, which partly relieved the pain in the head. She continued her attendance as an out-patient for about four months, subsequently to which an exfoliation of bone took place, and she applied at St. Bartholomew's Hospital, where Mr. Earle cut down upon the extremity of the stump. She returned to this hospital without any diminution of her sufferings; and after attending a short time, Mr. Luke having ascertained that the pain proceeded from that part of the stump where the median nerve appeared to terminate, proposed to remove a portion of the nerve, to which operation she consented.

April 21st—Mr. Luke made an incision in the course of the median nerve just below the axilla; and, after a little dissection, exposed and removed nearly half an inch. The woman thought that something was pulled, and complained of a sensation of numbness at the extremity of the stump: she immediately expressed herself entirely relieved from the pain, and could bear firm pressure upon it. In performing the operation, a small artery was divided, which required a ligature."

Some pyrexia succeeded and required saline medicines and venesection. Retention of urine succeeded, and she requires to have her water drawn off twice daily. The last report is dated June 12th, not two months from the performance of the operation, and it states that the wound is healed, that she is entirely free from pain, but that the retention of urine continues.

Now there are two considerations which induce us to think the reporter premature in giving this as a case of cure. In the first place, the period passed without pain, after the amputation performed by Mr. Langstaff, was longer than what has elapsed between the report and the operation on the median nerve, and yet we have seen that the former pause was deceptive, and that the disease returned. In the second place it seems evident to us from several circumstances, that the young woman is of an excitable, nervous, or hysteric habit, and we all know how difficult it is to treat such with success. We do not say that the above was a case of hysteria only, but we do say that there is much of that undefinable state of system mixed up with it, and that probably surgical operations will not put that out. However, be this the fact or be it not, we conceive the reporter hasty in publishing the case as one of cure.—*ib.*

24. *Diffused Hydrocele of the Cord.*—We have lately seen two specimens of hydrocele of the cord, which can hardly be considered examples of that which is usually called encysted. The instances of encysted hydrocele of the cord, which we have seen, have been small, more or less globular in form, and circumscribed. But the swelling in the cases we are about to describe had not these characters.

We were struck with the appearance of the scrotum of a physician's patient in the dead-house of the hospital. There was a swelling, which, at first sight, looked like that of common hydrocele. It was large, and extended from near the ring to near the bottom of the scrotum—pyriform, with the fundus below—immoveable, fluctuating, and *very* transparent. The testis was at its bottom, and rather to one side, but was firmly attached to the swelling. On dissecting the tumor, the cord was found spread out, its veins, in particular, being scattered irregularly over the sides of the swelling. The cyst was very thin, and occupied the cord from just below the ring to the testis; it appeared to be rather formed from the interstitial cellular tissue of the cord, than from that general cellular envelope or fascia which surrounds it. It was firmly attached below to the epidydimis, the direction of which was nearly horizontal, instead of obliquely from above downwards. The testis was below instead of in front, and its malposition gave it a dislocated appearance; it was healthy, and the cavity of the tunica vaginalis, though large, was free from fluid. The fluid of the cyst was very pellucid.

This may be considered as merely a large encysted hydrocele of the cord. However that may be, it is well for practitioners to know that the ordinary descriptions of encysted hydrocele will not apply to it, and that its characters so nearly resemble those of common hydrocele, as to make it likely to be mistaken for it. The points in which it differs from common hydrocele are, the position of the testis below rather than behind, and its very pellucid appearance between the eye and the light. Such a cyst, it must be recollected, is not strictly a serous membrane, nor so prone to inflammation and obliteration of its cavity by adhesions. It is well known that injections will not cure encysted hydrocele of the cord as they will hydrocele of the vaginal tunic, and it is probably from the operation of this law, that they will not do so. On looking at one of these cysts, we usually observe that it is extremely thin, and possesses but little vascularity; its growth is slow, its inflammation weak. In the encysted hydrocele of the cord, a seton will sometimes effect a cure—incision, and the introduction of lint into the cyst, is more certain. The treatment, which, when applied to the fibro-serous tunica vaginalis, excites very violent inflammation, causes barely sufficient in the pellucid cysts of the cord. It is on this account that we have drawn attention to this form of hydrocele, that practitioners may not deceive themselves and disappoint their patients, from imagining that they have a common hydrocele to deal with, when one of the kind we have been describing presents itself. Surgeons in large practice are probably well acquainted with the subject; but all surgeons are not in large practice, and things familiar to a Cooper or a Brodie are far from familiar to them.—*ib.*

25. *Scrofulous Disease of the Bones.*—Case 1. A young girl had long labored under scrofulous disease of the shoulder joint; it was much swelled, and covered with numerous ash-colored ulcers, from which flowed a profuse discharge—there were several fistulæ running towards the head of the humerus; but the bone could not be detected bare, by the probe. The joint was anchylosed; and the limb quite wasted—diarrhœa and hectic fever had brought the patient to death's door. The constitutional symptoms were first corrected; and then the use of iodine commenced, externally in the form of baths thrice a week; and the shoulder was daily rubbed with an ointment of ioduret of lead. As the patient bore the use of the iodine well, its inward use was now employed, and two ounces of the mineral iodated water given, the dose being gradually increased to eight and twelve ounces. The fistulæ were injected with a solution of iodine. The beneficial effects were speedily obvious; the joint was much reduced in volume, and the suppuration diminished in quantity. After the iodine had been taken for nearly two months, its use was suspended for three weeks, and diluents and aperients administered, with the view of counteracting any pernicious effects which the drug might have on the stomach, or on the general health; but again resumed and continued for six to eight weeks. In this way the medicine may be exhibited for a great length of time without any danger, especially if the preparation given is the solution of the hydriotate of ioduretted potass (a grain and a half of iodine, and three grains of hydriodate of potass in the course of the day.)

This patient obtained the cure of all the sores, and the state of the joint, though necessarily remaining anchylosed, was vastly improved.

Case 2. A child aged seven presented the left shoulder much puffed and enlarged; numerous fistulæ opened over the head of the bone, which was certainly much enlarged in size; the limb was quite atrophied—the motions of the joint were almost entirely lost—he had cough, diarrhœa, and evening fever. Mild nourishing food was ordered; the mineral iodated water given inwardly, and the shoulder rubbed with the ointment of ioduret of lead. In a fortnight the child was much improved; and to the above treatment was now conjoined frequent bathing in a river. The fistulæ were injected with a lixivial wash. At the end of three months, the movements of the joint had become much more free; the shoulder was very little swelled, and the limb had acquired flesh and strength. The iodine had been omitted at two periods, during which diluents and an occasional aperient were administered. The good effects were well marked in this case; the disease had not existed sufficiently long to expect a spontaneous cure; many anti-scrophulous remedies had been previously tried, and had all failed; but the iodine treatment had not been continued above a week or two, till the little patient shewed signs of improvement. These two cases are illustrative of strumous enlargement of the osseous texture. We shall now say a few words on the subject of caries. This morbid state most commonly attacks the small bones, as the phalanges of the toes and fingers, the metacarpal and metatarsal bones, and those of the tarsus and carpus. It is not rare in the vertebræ, or even in the sternum, ribs, and other long bones. Sometimes, but not always, it is preceded by swelling of the periosteum, or the bony structure itself. A dull, deep-seated pain without any appreciable cause, or it may be, supervening after a lapse of time to a contusion, is the earliest symptom; to this is soon added a general tumefaction, and agglutination of the surrounding soft parts. An abscess at length is formed and discharges a thin greyish matter mixed with albuminous flocculi, and sometimes with portions of bone. The probe will often detect the roughness of the exposed bone; and in a few cases will penetrate into the cavity of a joint; the existence of such a fistula may be also inferred from the admixture of synovial fluid with the discharge. M. Baudelocque, the author of this memoir, states that he has exhibited iodine in upwards of 30 cases of scrophulous caries of the bones; but seldom with any decided success, amounting to a cure. Its action, however, was very obviously as beneficial to a certain extent in these, as in any other examples of strumous disease; but since morbid changes in bones are more slowly formed, and more slowly advance, than in softer textures, so the cure must necessarily be more tedious and protracted. A period of six months, and upwards, will be generally found necessary; and it is seldom that we can have patients, especially in public institutions of charity, submitted to treatment for such a length of time.—*Revue Medicale.*

26. *Puerperal Peritonitis, followed by Ascites and Spontaneous Perforation of the Abdominal Parieties.*—F. G. agad 30, was delivered April, 1830, of her 7th child. On the second day after her confinement she was much agitated; and the consequence was, that severe pains in the abdomen came on, the lochia were suppressed, and the mammæ became flaccid. The symptoms were alternately relieved and aggravated for eight days; and at this period Dr. Puntons was called in. He found the patient much dispirited and alarmed; belly distended, sonorous, and painful on pressure; distinct fluctuation; suppressed lochia; urine much diminished in quantity, obstinate constipation, severe headache, tongue clean, much thirst, pulse irregular, somewhat hard and very frequent; œdema of the feet and ankles.

*Treatment.* Emollient fomentations, mercurial frictions on the inside of the thighs, calomel and nitre inwardly. Diet, chicken broth. This plan of treatment was persevered in for a week; the abdominal tenderness had much abated; but the distention had become greater; pulse less hard—a blister ordered to be applied to each thigh. On the 18th day from the commencement of the attack, an opening or slit occurred in the abdominal parieties, about an inch below the umbilicus; the part had been smooth and glistening for a few days before. About a pint of yellowish, inodorous fluid escaped. Two days afterwards a similar vent took place in the upper part of the left hypogastric region, and an ounce or two of a citron colored serum flowed out. Simple dressings applied to the wounds, and firm compresses to the abdomen, in order to promote the expulsion of the fluid. The woman gradually recovered.

*Observations.* An analogous case is mentioned by Frank. The effused fluid was visible through the navel. The author of the Nosographie Philosophique narrates an example of puerperal peritonitis, in which a large abscess formed in the left labium; on the 14th day, it broke, and the patient was speedily cured. Van Swieten and Chomal allude to many cases in which the abscesses had found their way into the intestines and caused a purulent diarrhœa.—*ib.*

27. *On large doses of Quinine in Atmospheric Fevers.* By H. Perrine, M. D. (Extracted from a letter to William P. Dewees, M. D.)—Laboring under the symptoms of *cholerine* on the eve of my expected return to Campeche, I cannot depart from this *stage* or *state* without renewing my testimony of the great virtues of the sulphate of quinine. I commenced the practice of medicine in Illinois, in the fall of 1819, contined it in that state until the spring of 1824, and in the state of Mississippi until the spring of 1826, when I embarked at New Orleans on account of my health for Cuba, and thence proceeded through Boston to Canada during the summer. The following winter was passed in this city. In May, 1827, I sailed for Tobasco, in Mexico, where I remained during the rainy season, and arrived at my consulate in Campeche of Yucatan, in November. I returned to Tobasco in July, 1830, and sailed from that port for this city in June, 1831, and in September last was again at Great Sodus bay in this state where I had been a short time in 1826. You will hence perceive that I have had opportunities for medical observation in various degrees of latitude and longitude between the southern shores of our great lakes and the northern base of our Guatemalian mountains.

During the first six years of my practice I used and recommended with gradually increasing boldness, *large doses* of the Peruvian bark, *frequently repeated* during the *paroxysms of fevers.* My communication in your Journal of November, 1826, contains my first essays with *large doses of the sulphate of quinine.* I have since had abundant opportunities of witnessing their effects in the hands of myself, of other physicians, and of *the people.* In 1827, quinine was first known in Tobasco and Yucatan, and was bought by *physicians* at two shillings a *grain.* I introduced its use in large doses, during the febrile paroxysms. In 1831, it was bought by *families* at five dollars an *ounce.* Every person that adopted my practice, has never, I believe, abandoned it. I therefore merely claim common capacity for observation and common veracity for report in submitting very briefly a few general results of my experience:

1. The medium dose of the sulphate of quinine at any period of fever from its incipient to its terminating symptoms is *ten grains* to be repeated every *two hours,* whatever be the state of the pulse and skin.

2. It may be given without interfering with simultaneous antiphlogistic stimulant, or other auxiliary measures according to symptoms, any more than an equal quantity of James' powder. Indeed if the quinine should be slily substituted in the paper, the physician would express his surprise at the immense sudorific and sedative powers of this new pulvis antimonialis.

3. The retreat of fever under the power of quinine as under the ower of nature, is indicated by the changes of the hot or the cold skin, and of the strong or the feeble pulse towards their natural condition; and most generally by secretion from the skin, often from the kidneys, occasionally from the bowels, and sometimes from *all three in succession.*

4. The *general* disturbance of the nervous and vascular systems, called febrile, whether cold and depression or heat and excitement be the alarming traits, may be counteracted on the first day by at most six doses, or on the second by a dozen; and thus will be prevented that gastro-enteritis,, a *consequence* of endemic or epidemic fever which Broussais has mistaken for the *cause.*

I have hence reason to believe that all malignant fevers, (even if called the *cholera* when their force is attracted to previously disordered bowels,) may be cut short at their commencement by large doses of sulphate of quinine; and that local disorder may at the same time or afterwards be corrected by the same means that were effectual for the same symptoms before the attack.

I am happy to see in Magendie's Formulæ, that large doses of quinine are administered by highly reputable physicians of Europe.

*New York, June* 26, 1832.

28. *Obituary Notice of Dr. Spurzheim from the Funeral Oration of Professor Follen.*—"Gaspar Spurzheim was born on the 31st December, 1775, at Longvich, a village about seven miles from the city of Treves, on the Moselle, in the lower circle of the Rhine, now under the dominion of Prussia. His father was a farmer, and in his religious persuasion, a Lutheran. Young Spurzheim received his classical education at the College of Treves; and was destined by his friends, for the profession of Theology. In consequence of the war between Germany and France, in 1797, the students of that college were dispersed, and Spurzheim went to Vienna. Here he devoted himself to the study of medicine, and became the pupil, and afterward the associate of Dr. Gall, who was at that time established as a physician at Vienna.

"This extraordinary man had been induced, by an observation made by him when a boy of nine years old, to attempt a new mode of scientific investigation. While at school, young Gall felt mortified at seeing himself surpassed by a number of his school-fellows in all those exercises that required verbal memory. The mortified pupil tried to find out some reason to account for this fact, that boys who in their other exercises were much his inferiors, yet showed better heads in committing lessons to memory. He was struck with the observation hat those boys who learned so easily by heart, had remarkably large and prominent eyes. The connexion of this external sign and that mental faculty occurred to him, and he inferred that prominent eyes were a mark of good memory. This observation, insignificant in itself, led Gall to study minutely, on the one hand the prominent talents and individual characters of men, and on the other hand the form of their heads. Little by little he flattered himself that he could perceive one constant shape in the head of every great painter, every great musician, every great mechanic, severally denoting a decided predisposition in the individual to one or the other of these arts. The study of medicine, and particularly of anatomy, to which he devoted himself, soon induced him to consider the peculiar form of the skull only as the evidence and the effect of that of the brain. This part of the human body, which had always been considered as the principal instrument of the mind, became now the chief object of Gall's investigation; and instead of considering the whole brain, as was formerly the case, as required for each of the different manifestations of the mind, he examined each part with special reference to those prominences of the skull which he had before perceived to be indications of particular talents and dispositions. He considered each of these parts of the brain as the particular organ of each of the different faculties of the mind, in the same manner as the eyes are the organs of sight, and the ears of hearing. Thus, from two sources of observation, from the study of the variety of talent and character, and of the organization of the brain, there arose a new science, the Physiology of the brain, that is, the theory of the different parts of the brain considered as the organs or instruments of the various animal, intellectual and moral capacities.* The physiology of the brain which at first frequently went by the name of Craniology, or the doctrine of the skull, is now more generally known by that of Phrenology, or the doctrine of the mind, by which Spurzheim preferred to designate this new science.

" It was at Vienna, in the year 1800, that Spurzheim first attended a private course which Dr. Gall had repeated from time to time, during the four preceding years, in order to explain to a select audience his new theory of the organs and functions of the brain. The dissections of the brain itself still remained imperfect until 1804, when Spurzheim became his associate, and undertook especially the anatomical department. From that time, in their public as well as private demonstrations of the brain, Spurzheim always made the dissections, and Gall explained them to the audience.

"The great interest which was excited by these lectures at Vienna, and throughout Germany, roused the fears of that inveterate enemy of all innovations, the government of Austria. An imperial decree, which prohibited all private lectures unless by special permission, silenced the two teachers, and induced them, in 1805, to quit Vienna. They travelled together through Germany, explaining and demonstrating their physiological discoveries in the principal universities and cities; particularly in Berlin, Leipzig, Dresden, Halle, Heidelberg and Munich. Their anatomical demonstrations excited every where great interest and applause. The great German anatomist and physiologist, Reit, before whom Gall and Spurzheim dissected a brain in 1805, at Halle, said to Professor Bischoff, who wrote an exposition of their

*Hence the medal that was published at Paris after Gall's death, in 1828 bears the inscription, 'Au createur de la physiologie du cerveau.' (To the author of the physiology of the brain.)

doctrine, 'I have seen in the anatomical demonstrations of the brain, made by Dr. Gall, more than I thought that a man could discover in his whole life.' Their peculiar physiological doctrines on the organization of the brain being adapted to various innate qualities of the mind, found many opposers, but also some warm adherents, and gave rise to a great number of publications in which the subject was discussed.*

"In the year 1807, Gall and Spurzheim went to Paris, where they demonstrated their theory of the brain in the presence of Cuvier, then the chief of the anatomical department of the French Institute, and before many other distinguished men, and learned Societies.

"In Paris, Gall and Spurzheim began to publish their great work on the anatomy and physiology of the nervous system in general, and that of the brain in particular. They also continued their public lectures and demonstrations. They remained and labored together in Paris till the year 1813. In the following year Spurzheim went over to England, and gave his first public lecture in London, in the amphitheatre of Mr. Abernethy. Mr. Abernethy, though he did not give full credit to the evidence brought forward by phrenologists, to prove that special parts of the brain are the organs of certain innate qualities of the mind, fully acknowledged the superiority of Dr. Spurzheim's anatomical demonstrations over every previous mode of dissecting the brain.

"He visited England again in 1825, and was engaged partly in lecturing, and partly in the publication of different books. The first work he had published in England, 'The Physiognomical System,' contained several summary views of different branches of anthropology, which he now endeavored to make more generally appreciated, by extending the principal chapters, and making them separate books. In one of them, *Phrenology*, he treats of the different powers of the mind, and their cerebral organs, in general. A smaller book, *Outlines of Phrenology*, is an abstract of that work. The two principal doctrines of Phrenology, that of the brain, and that of the mind, were carried out in different works.

"In his *Anatomy of the Brain*, he laid down his and Gall's investigations of the brain and the nervous system. On the other hand, the doctrine of the mind, with its practical bearings on religion and morality is carried out in his *Philosophical Principles of Phrenology*. The same principles, in a more condensed and practical form, are set forth in his *Philosophical Catechism of the Natural Laws of Man*. The subject of education, on which he rested all his philanthropic hopes, was treated of in his *Elementary Principles of Education*, a book full of the most important information, and excellent counsel. The deranged functions of the brain is the subject of his interesting work on *Insanity*, for which his frequent visits at the Insane Hospitals

* The first expositions of the doctrines of Gall and Spurzheim were published in Germany between the years 1802 and in 1805, by Froriep and Walter. Bischoff, Knoblauch, Bloede; and in Paris, in 1806, by Demaugeon.

afforded him a great number of important observations. All the works which Dr. Spurzheim edited after his separation from Dr. Gall in the year 1813, show a spirit of free and indefatigable inquiry. The improvement in the anatomy of the brain, was chiefly Spurzheim's work; he also discriminated more minutely between different faculties of the mind which Gall had confounded, and he endeavored to point out their relation to the development of the brain; he moreover brought method and order into the scattered doctrines of Phrenology."

In education he was anxious that the moral and physical cultivation of the pupil should not be sacrificed to the intellectual, and he thought many of our establishments for education deficient in these respects. In his choice of duties, he "always chose for himself in preference, the performance of that duty which required the greatest effort and self-denial;" and he adds, what it gives us much pain to learn, "that his anxious desire to fulfil his engagements in Boston and in Cambridge, was the chief cause of his death. Although oppressed by indisposition and contrary to the entreaties of his medical friends, he continued to lecture; and once in his last sickness, he started up with the intention to dress himself to go to Cambridge." At the close of each lecture, he listened patiently to every inquirer, however humble, and never turned him by, to attend to the great, who were waiting.

He never suffered poverty to exclude any one from his lectures, and when his friends were his almoners in the distribution of free tickets, he wished never to be informed to whom they were given.

His love of truth was supreme; he wished no one to believe any thing on his authority but simply from conviction, after due examination. He was unwilling that phrenology should become an instrument of soothsaying and quackery, and he always refused to designate the characters of living individuals by the application of the rules of his science.

He did not believe, with Dr. Gall, that there was an organ for theft and one for murder, which he thought inconsistent with the benevolence of God.

"All his writings and lectures were marked by the decidedly religious tendency of his mind. He firmly believed in the essential truths of natural and revealed religion. He adopted christianity as a divine system, chiefly on the ground of its great internal evidence, its perfect adaptation to human nature, and the spirit of truth and divine philanthropy which gives life to all its precepts. All morality, he thought, was contained in two precepts—'Thou shalt love the Lord thy God and thy neighbor as thyself.' All prayers, were comprised in this one—'Father, thy will be done.' "

"The great aim of all his inquires was to search out the will of God in the creation of man. Obedience to his laws he considered as the highest wisdom and the most expansive freedom," included in the short sentence, 'Thy will be done.' "We look to him perhaps," he would say, "amid great trials and on great occasions; but not in smaller things. We say, 'they are too little.' It is this, in which we err. Can any thing that concerns his children be too little for a *Father*?"

Through his sickness, however, he was patient and submissive, and distinctly announced his own views of his case in the emphatical words "I must die;" when his friend replied—I hope not—he added "Oh yes, I must die; I wish to live as long as I can, for the good of the science; but I am not afraid of death."

With his hands folded upon his breast, and an uplifted countenance, he died without a groan, on the night of the 11th of November; and although he breathed his last in a land of strangers, he was surrounded by affectionate friends, and by able physicians, emulous of doing something to rescue him from death, or to smooth his downward passage to the grave. He was in the fifty-sixth year of his age.

